# Protamine nanoparticles for improving shRNA-mediated anti-cancer effects

**DOI:** 10.1186/s11671-015-0845-z

**Published:** 2015-03-19

**Authors:** Ming Liu, Bo Feng, Yijie Shi, Chang Su, Huijuan Song, Wei Cheng, Liang Zhao

**Affiliations:** School of Pharmacy, Liaoning Medical University, Jinzhou, 121000 People’s Republic of China; School of Veterinary Medicine, Liaoning Medical University, Jinzhou, 121000 People’s Republic of China; Central Laboratory of Liaoning Medical University, Jinzhou, 121000 People’s Republic of China

**Keywords:** Protamine, Nanoparticles, Bcl-2, Transfection, Cytotoxicity

## Abstract

Protamine nanoparticles were designed by encapsulating small hairpin RNA (shRNA)-expressing plasmid DNA targeting the Bcl-2 gene (shBcl-2) to silence apoptosis-related Bcl-2 protein for improving the transfection efficiency and cytotoxicity in cancer therapy. Our findings demonstrated that the obtained protamine nanoparticles possessed excellent characterizations of small particle size, homogenous distribution, positive charge, and high encapsulation efficiency of gene. shBcl-2 loaded in nanoparticles (NPs) was protected effectively from the degradation of DNase I and serum. More importantly, it significantly improved the efficiency of transfection of shRNA *in vitro* in A549 cells and increased its cytotoxicity and induced more cell apoptosis by silencing Bcl-2.

## Background

Although traditional therapies such as surgery, radiotherapy, chemotherapy, and other traditional means can improve the curing effects in the early stage of cancer patients in some extent, it seemed unvalued to fight against metastasis, recurrence of tumor, and the treatment of advanced cancer. It was well known that some genes such as oncogenes and cell apoptosis-related genes including Bcl-2 were highly expressed in tumor cells and facilitated the cell unlimited reproduction and carcinogenesis [1-4]. Therefore, the cancer therapy based on silencing the specific gene such as Bcl-2 for killing tumor cells becomes a new and potential application in tumor treatment [5-9].

Nanoparticles as a gene carrier showed their special merits compared to other traditional drug delivery systems [10,11]. Firstly, it can efficiently encapsulate DNA, RNA, peptide nucleic acid (PNA), and double-stranded RNA (dsRNA) and prevent them from degradation by enzymes [12]. Secondly, duo to the small particle size, nanoparticles could easily escape from tumor vessels and be kept around tumor tissue for longer time known as enhanced permeability and retention effect (EPR), thus favoring the accumulation and distribution of drugs [13]. More importantly, nanoparticles as a whole were internalized into cells though endocytosis and avoided contact between gene and proteins situated at the surface of cells, leading to the significant improvement on the transfection efficiency of gene [14-16].

Protamine, as a kind of basic protein, acts on combining with DNA in fish in the mature sperm nucleus. The relative molecular mass of protamine is about 6,000 ~ 10,000 and consisting of 30 amino acid residues in which two thirds is the arginine. Toxicological experiments have proved its excellent biocompatibilities including nontoxicity, harmlessness, good stability, and heating solidification [17,18]. Interestingly, protamine contains a nuclear localization signal (NLS); it and its binding partners can be transported into the nucleus, improving the efficiency of exogenous substances into the cell nucleus [19,20].

In this work, protamine nanoparticles as a gene carrier were prepared by desolvation method and the mass ratio between gene and protamine was also optimized by determining their particle size, zeta potential, and morphology. The small hairpin RNA (shRNA)-expressing plasmid DNA targeting the Bcl-2 gene (shBcl-2) as a model gene was encapsulated in nanoparticles (NPs) to treat cancer more efficiently. The *in vitro* transfection efficiency, cytotoxicity, and western blot were investigated in lung cancer cell A549.

## Methods

### Materials

Protamine was purchased from Tianjin Sixth Pharmaceutical Co., Ltd (Tianjin, China), 3-(4,5-dimethylthiazol-2-yl)-2, 5-diphenyltetrazolium bromide and proteinase K were obtained from Sigma-Aldrich (St. Louis, MO, USA), and pGCsi-U6/Neo/GFP-Bcl-2 shRNA-expressing plasmid DNA (pDNA) (Bcl-2 shRNA, shBcl-2) which targeted Bcl-2 mRNA sequence (GATGAAGTACATCCATTAT) and pGCsi-U6/Neo/GFP-shRNA-expressing pDNA (pEGFP) were purchased from Genechem Co., Ltd. (Shanghai, China). All other chemicals purchased were of analytical grade and were obtained from a variety of vendors. A549 cells were obtained from Liaoning Medical University.

### The preparation and characterization of shBcl-2-loaded protamine NPs

Certain amount of protamine was swelled with distilled water overnight to obtain an aqueous solution containing protamine at 1.5, 2.5, and 4 mg/mL. After that, the shBcl-2 was also added into the obtained protamine solution followed by the addition of 1 mL of ethanol at 37°C under continuous stirring (1,000 rpm). The resulting white suspension was further stirred for 24 h, and then ethanol was removed with vacuum distillation succeeded by the addition of 8% glutaraldehyde in water (0.5 μL per mg of protamine) for particle cross-linking. After washing with cold PBS three times, the resulting nanoparticles with different mass ratio of protamine and shBcl-2 (50:1, 100:1, and 200:1) were purified and sterilized by filtration using Millex GP Filter Unit (0.22 μm, Millipore, Billerica, MA, USA). The characterization of shBcl-2-loaded NPs was investigated to determine the particle size and zeta potential by using dynamic light scattering (DLS) (NanoZS, Malvern Instruments, Malvern, UK), and the morphology of particles was observed by using transmission electron microscope (TEM) (Jeol, Tokyo, Japan). The difference between the amount of the initially added shRNA and shRNA in the supernatant was measured at detecting absorbance at 260 nm using a UV/Vis spectrophotometer (model 1601, Shimadzu, Kyoto, Japan) to determine the encapsulation efficiency (EE) of shRNA in nanoparticles.

### Gel retardation experiments with shBcl-2-loaded NPs

The obtained NPs were collected and the shRNA in the supernatant was removed by centrifugation at 16,000 rpm. The collected NPs were degraded by incubating with a medium containing 400 μL of cell lysates, 200 μL of SDS (10%), and 100 μL of proteinase K (Sigma-Aldrich) solution (20 mg/mL) at 57°C overnight, and shRNA loaded in NPs was extracted with phenol-chloroform-isoamyl butyrate method and analyzed by agarose gel electrophoresis.

### DNase I protection test

The collected NPs were added into 10 μL of buffer containing 2 U of DNase I and replenish to 50 μL with nuclease-free water for continuous incubation at 37°C for 1 h. As 1 μL of stop buffer was added into the medium to stop the reaction, shRNA from NPs was extracted according to the above method and the products were analyzed by agarose gel electrophoresis. At the same time, the naked shRNA was treated by the same method as a control treatment.

### Serum protection test

NPs were immersed in cell culture medium containing 10% fetal bovine serum and continue to incubate at 37°C for 8 h. NPs were collected and shRNA was extracted according to the procedure described in ‘Gel retardation experiments with shBcl-2-loaded NPs’ section and were analyzed by agarose gel electrophoresis. At the same time, the naked shRNA was also treated by the same method as a control treatment.

### *In vitro* transfection experiments

Enhanced green fluorescent protein (EGFP) was used as the reporter gene to compare the transfection efficiency of free EGFP and EGFP-loaded NPs. After adherence of A549 cells at a density of 5 × 10^4^/mL, free EGFP and EGFP-loaded NPs containing the same concentration of EGFP were added into the culture medium and incubated with cells for 48 h. After that, the supernatant was discarded after centrifugation and cells in well were washed with ice-cold PBS for three times for the observation of their images with fluorescent microscopy (DMI400B, Leica Microsystems, Wetzlar, Germany).

### *In vitro* cell viability assay

A 3-(4,5-dimethylthiazol-2-yl)-2, 5-diphenyltetrazolium bromide (MTT, Sigma-Aldrich) assay was used to compare cell inhibiting effects in A549 cells treated with free shBcl-2 and shBcl-2-loaded NPs. Cells were seeded into the 96-well plate (100 μL each well) for cellular adherence at 37°C under 5% CO_2_ and 95% O_2_. After that, free shBcl-2 and shBcl-2-loaded NPs at different amounts were separately incubated with cells for 24 h followed by the addition of 20 μL MTT with concentration of 5 mg/mL and continued to be incubated for 4 h at 37°C. Then, the supernatant was aspirated, 150 μL of DMSO was added to each well, and the absorbance was measured at 490 nm using a microplate reader (iMark™, Bio-Rad, Hercules, CA, USA).

### Western blot assay

Cells were treated with free shBcl-2 and shBcl-2-loaded protamine NPs and washed twice with ice-cold PBS, and then lysed in RIPA buffer (150 mM NaCl, 1% NP-40, 1% SDS, 1 mM PMSF, 10 μg/mL leupeptin, 1 mM aprotinin, 50 mM Tris-Cl, pH 7.4). About 50 μg protein in 20 μL of cell lysate separated by 10% SDS-PAGE was transferred onto polyvinylidene fluoride (PVDF) membrane. After treatment with 1% BSA for blocking, the primary antibodies (Bcl-2, caspase-3, poly ADP ribose polymerase (PARP)) were incubated with the PVDF membrane at 4°C, overnight, followed by continuous incubation with secondary antibody for 1 h and stained with ECL. The levels of the targeted proteins were photographed and analyzed by UVP gel analysis system (iBox Scientia 600; UVP, LLC., Upland, CA, USA).

## Results

### The characterization of shBcl-2-loaded NPs

**Figure 1:**
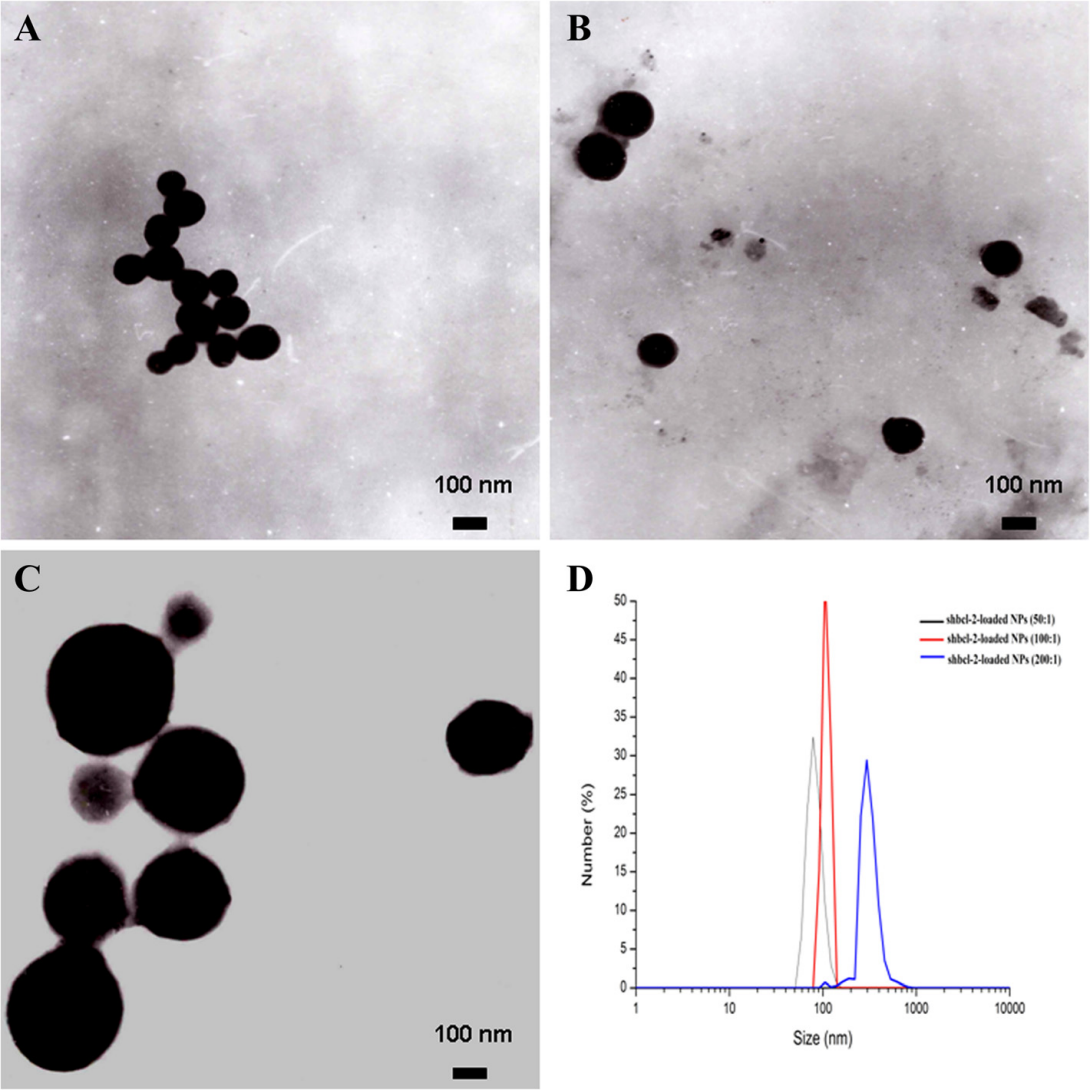
**TEM images of NPs.** shBcl-2-loaded NPs (50:1) **(A)**, shBcl-2-loaded NPs (100:1) **(B)**, and shBcl-2-loaded NPs (200:1) **(C)**. DLS analysis of the obtained shBcl-2-loaded NPs **(D)**.

The morphology and size distribution of the prepared nanoparticles were determined by TEM and DLS, and the results were displayed in Table 1. It can be seen from Table 1 that with the increasing amount of protamine, the particle size was enhanced and the zeta potential also continued to rise. In term of encapsulation efficiency of shBcl-2 in NPs, about 85% of shBcl-2 was loaded in NPs with the mass ratio at 100:1 in contrast with the other two NPs. Judging by the TEM in Figure 1, it was observed that shBcl-2-loaded NPs with different mass ratios possessed an excellent characterization of suitable particle size, homogenous size distribution, and high monodispersion, indicating that shBcl-2-loaded NPs with the mass ratio of 100:1 was a potential optimized gene carrier with good characterization and higher encapsulation efficiency.

**Table 1:** **Key parameters of shBcl-2-loaded NPs**

**Group**	**Diameter (nm)**	**Zeta potential (mV)**	**Polydispersity**	**Encapsulation efficiency (%)**
shBcl-2-loaded NPs (50:1)	82 ± 9	+5.67 ± 0.94	0.21 ± 0.07	60.98 ± 2.46
shBcl-2-loaded NPs (100:1)	108 ± 15	+10.32 ± 2.67	0.15 ± 0.08	85.42 ± 3.87
shBcl-2-loaded NPs (200:1)	298 ± 30	+13.68 ± 3.43	0.20 ± 0.04	68.78 ± 4.67

### Stability and protecting effects of shBcl-2-loaded NPs

**Figure 2:**
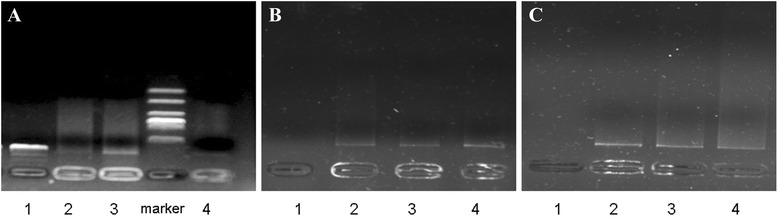
**Combining ability and protecting effects of shBcl-2-loaded NPs by agarose gel electrophoresis.** Gel retardation assay for determining loading ability of shBcl-2 in NPs **(A)**, DNaseI protection of shBcl-2-loaded NPs **(B)**, and serum protection of shBcl-2-loaded NPs **(C)**. (Lane 1: naked shBcl-2; lanes 2 to 4, shBcl-2-loaded NPs prepared at mass ratios of 50,100, and 200, respectively).

Positive-charged NPs can encapsulate negative-charged shRNA owing to the strong electrostatic attraction. With the increase of amount of shRNA in NPs, the electrophoretic mobility of DNA is faster and the band will be stronger. The results in Figure 2 showed that when the ratio of protamine and DNA was 100:1, the binding ability of shRNA with NPs was the strongest among the three groups, suggesting that more shRNA was entrapped in NPs.

In order to investigate the protection of NPs from the degradation of shRNA, DNase I protection test and serum protection test were performed *in vitro*. The results showed that after the naked shRNA was digested by DNaseI for 1 h or incubated with fetal bovine serum for 8 h, the band disappeared suggesting that the naked shRNA was degraded thoroughly. In contrast, after digestion, the band of shRNA loaded in NPs still remained, implying that nucleic acid in NPs was not degraded and retained the integrate structure. It suggested that shRNA was efficiently protected from the enzyme’s degradation by encapsulation in NPs.

### Cytotoxicity

**Figure 3:**
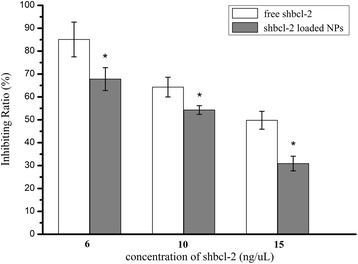
**Viability of A549 cells after treatment with free shBcl-2 and shBcl-2-loaded NPs.** Viability of A549 cells after treatment with free shBcl-2 and shBcl-2-loaded NPs with the concentrations of shBcl-2 at 6, 10, and 15 ng/μL. *P < 0.05, vs the free shBcl-2 group treated with A549 cells.

The inhibiting effects of free shBcl-2 and shBcl-2-loaded NPs on the amplification of A549 cells were determined by MTT assay. It can be seen from Figure 3 that shBcl-2-loaded NPs showed higher cytotoxicity at different concentration than free shBcl-2, suggesting that more shBcl-2 in NPs were transported into cells with the mediation of internalization of NPs. It also indicated that different from penetration of free drug into cells, cellular uptake of shBcl-2-loaded NPs may depend on endocytosis and macropinocytosis and induced the sufficient internalization of NPs in cells. Therefore, cytotoxic effects of shBcl-2-loaded NPs were higher than those of free shBcl-2.

### *In vitro* transfection effects

**Figure 4:**
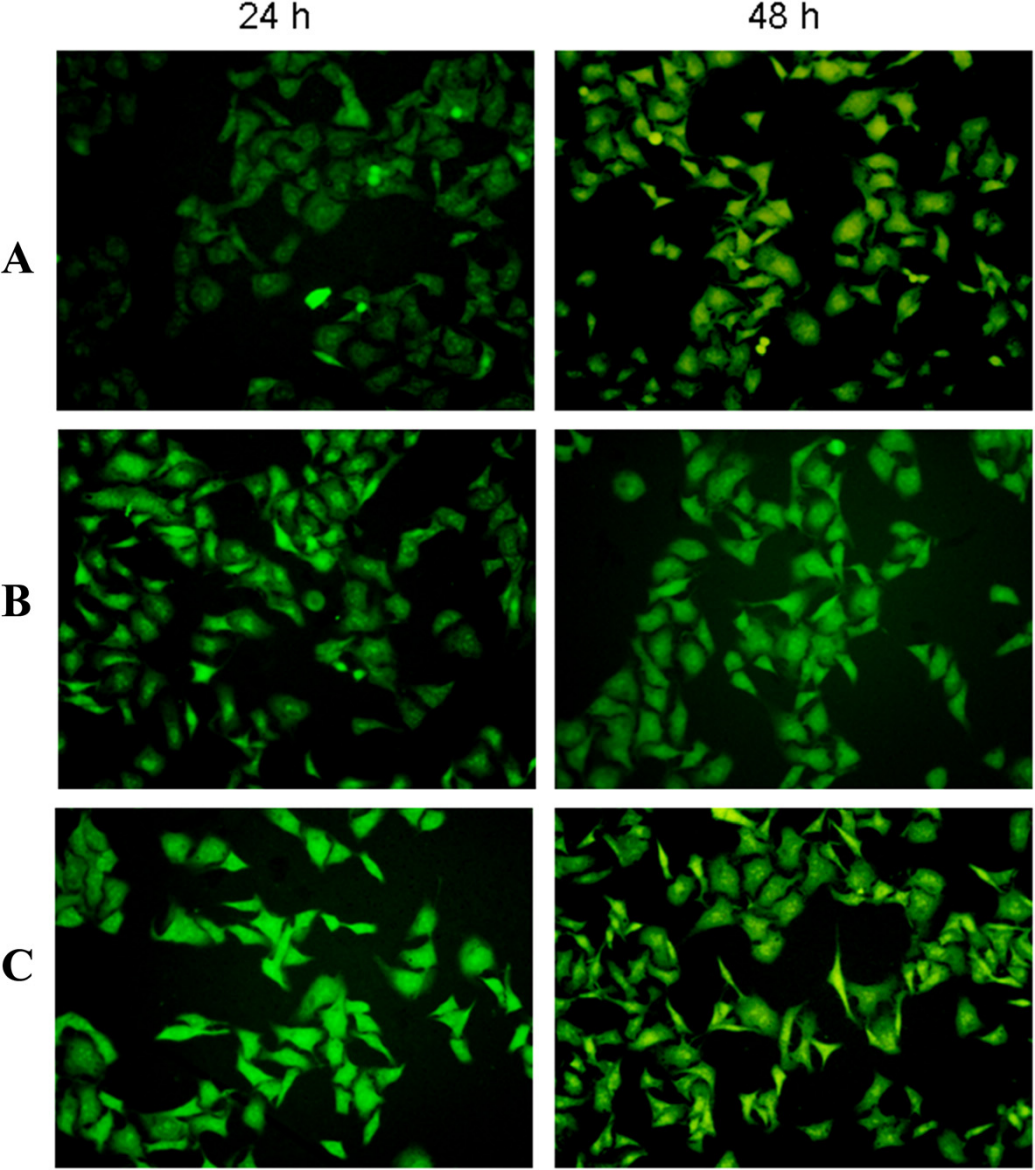
***In vitro*** **transfection efficiency of EGFP-loaded NPs.** *In vitro* transfection efficiency of EGFP-loaded NPs was investigated by observing the microscopic images of A549 cells after incubation with free EGFP and EGFP-loaded NPs within 48 h (**(A)** free EGFP at 2 μg each well, **(B)** NPs loading EGFP of 2 μg each well, and **(C)** NPs loading EGFP of 4 μg each well).

To evaluate the *in vitro* transfection effects of NPs, EGFP-loaded NPs were observed using the fluorescent images of cells. The images (Figure 4) showed that compared with free EGFP, cells treated with EGFP-loaded NPs showed the stronger green fluorescent color within 48 h. In addition, with the increase of the amount of EGFP in NPs, the intracellular green fluorescence intensity was increased. It demonstrated that the *in vitro* EGFP entrapped in NPs facilitated the cellular accumulation and avoided the extracellular degradation, thus leading to significant improvement on the *in vitro* transfection effects.

### Western blot analysis

**Figure 5:**
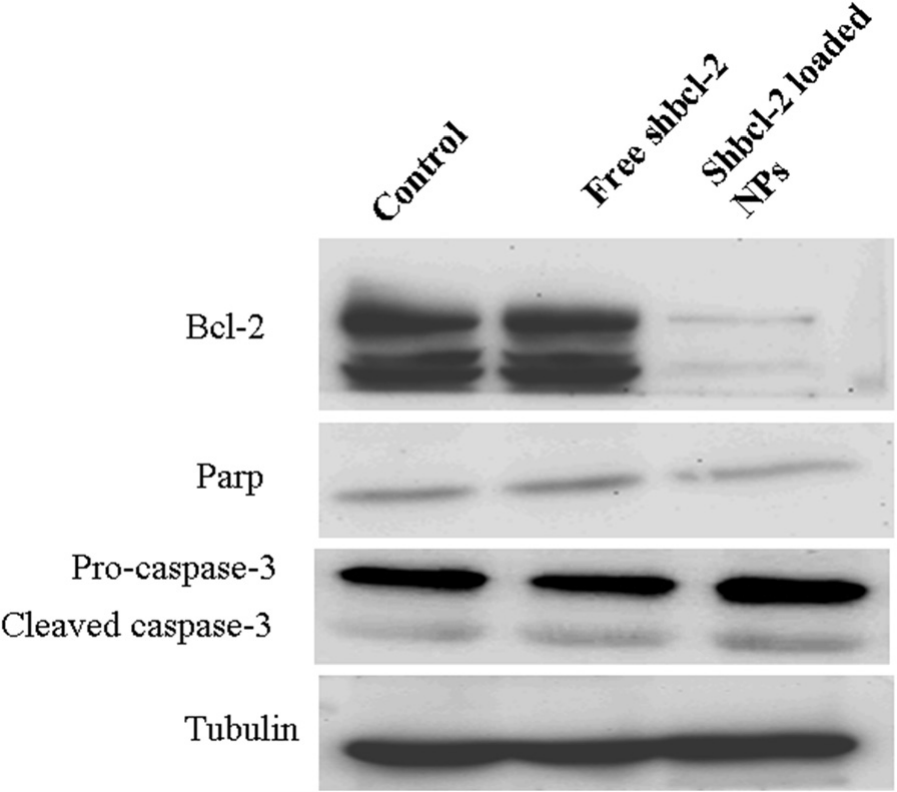
**Western blot analyses of the expression levels of Bcl-2, caspase-3, and PARP proteins.** Western blot analyses of the expression levels of Bcl-2, caspase-3, and PARP proteins in A549 cells after incubating with free shBcl-2 and shBcl-2 loaded NPs.

It can be seen from Figure 5 that compared with control and free shBcl-2, shBcl-2-loaded NPs inhibited the expression of Bcl-2 proteins significantly in A549 cells. We also detected the PARP and caspase-3 as the main apoptosis proteins to evaluate the apoptosis effects of cells induced by silencing Bcl-2. It demonstrated that the expression of caspase-3 was higher in cells treated with shBcl-2-loaded NPs than in those treated with free Bcl-2, indicating that shBcl-2-loaded NPs induced the higher apoptosis of A549 cells by silencing the Bcl-2 proteins and increasing the expression of caspase-3.

## Discussion

Basing on the electric attraction between positive and negative charges, negative-charged shRNA with high water solubility tended to encapsulate into the core of protamine nanoparticles with the positive-charged one. Compared with the disadvantages of naked shRNA such as fast degradation, short half-life, and difficulty in penetrating the cell membrane owing to the negative charge, gene-loaded protamine nanoparticles with the positive charges facilitated the internalization of cells through endocytosis and macropinocytosis and enhanced the transfection of shRNA *in vitro* in A549 cells [21,22]. Moreover, shRNA entrapped inside of NPs was isolated from the enzymes in the blood and cytoplasm in the transmitting process, thus improving its stability and curing effects.

The occurrence of tumor may have originated from cell proliferation and excessive inhibited apoptosis of tumor cells; Bcl-2 protein as the founding member of the Bcl-2 family of regulator proteins played a big role on regulating cell death (apoptosis), by either inducing it (pro-apoptotic) or inhibiting it (anti-apoptotic). Exogenous dsRNA *in vivo* is cut into 21- to 23-nt small interfering RNA (siRNA) and bonds to the RNase complexes to form gene RNA-induced silencing complex (RISC) [23,24]. With the mediation of ATP, double-stranded siRNA was depolymerized into single strands and participated in the transcription of the gene homologous sequences, thus eventually leading to gene silencing. The result showed that compared with naked shBcl-2, shBcl-2-loaded protamine nanoparticles significantly inhibited the expression of Bcl-2 proteins, thus improving their cytotoxic ability and inducing the cell apoptosis.

## Conclusions

We used protamine as the material to prepare gene-carried nanoparticles and investigated its applicability and feasibility for improving shRNA-mediated anti-cancer effects. The mass ratio of protamine and gene was also optimized basing on their physicochemical and biological properties. The results showed that shBcl-2-loaded protamine nanoparticles with a mass ratio of 100:1 possessed the excellent characterization with suitable particle distribution, positive charge, and higher encapsulation efficiency of shRNA. Compared with naked shRNA, nanoparticles could effectively protect shRNA from being degraded by nuclease and serum. More importantly, it significantly improved the transfection of shRNA *in vitro* in A549 cells. shBcl-2-loaded protamine nanoparticles increased their cytotoxicity of A549 and induced cell apoptosis by silencing Bcl-2. Taken together, protamine nanoparticle could be a promising nonviral nanodevice for improving the targeting delivery of gene in cancer therapy.

## References

[1] Yokota J (2000). Tumor progression and metastasis. Carcinogenesis..

[2] Shin SY, Lee JM, Lim Y, Lee YH (2013). Transcriptional regulation of the growth-regulated oncogene α gene by early growth response protein-1 in response to tumor necrosis factor α stimulation. Biochimica et Biophysica Acta (BBA)-Gene Regul Mech.

[3] Mutlu P, Ural AU, Gündüz U (2012). Differential oncogene-related gene expressions in myeloma cells resistant to prednisone and vincristine. Biomed Pharmacother..

[4] Alistair MC, Brian JJL, Carl RW, Sofie S (2014). Gene expression profiling to define the cell intrinsic role of the SKI proto-oncogene in hematopoiesis and myeloid neoplasms. Genomics Data..

[5] Wang XW, Zhao JF, Huang JH, Tang HH, Yu SY, Chen YX (2012). The regulatory roles of miRNA and methylation on oncogene and tumor suppressor gene expression in pancreatic cancer cells. Biochem Biophys Res Commun..

[6] Chu R, Liu SY, Vlantis AC, van Hasselt CA, Ng EK, Fan MD (2015). Inhibition of Foxp3 in cancer cells induces apoptosis of thyroid cancer cells. Mol Cell Endocrinol..

[7] Tsouris V, Joo MK, Kim SH, Kwon IC, Won YY (2014). Nano carriers that enable co-delivery of chemotherapy and RNAi agents for treatment of drug-resistant cancers. Biotechnol Adv..

[8] Rossowska J, Pajtasz-Piasecka E, Ryśnik O, Wojas J, Krawczenko A, Szyda A (2011). Generation of antitumor response by IL-2-transduced JAWS II dendritic cells. Immunobiology..

[9] Kunze D, Wuttig D, Fuessel S, Kraemer K, Kotzsch M, Meye A (2008). Multitarget siRNA inhibition of antiapoptotic genes (XIAP, BCL2, BCL-X(L)) in bladder cancer cells. Anticancer Res..

[10] Fornaguera C, Grijalvo S, Galán M, Fuentes-Paniagua E, de la Mata FJ, Gómez R (2014). Novel non-viral gene delivery systems composed of carbosilane dendron functionalized nanoparticles prepared from nano-emulsions as non-viral carriers for antisense oligonucleotides. Int J Pharm..

[11] Choi KM, Jang M, Kim JH, Ahn HJ (2014). Tumor-specific delivery of siRNA using supramolecular assembly of hyaluronic acid nanoparticles and 2b RNA-binding protein/siRNA complexes. Biomaterials..

[12] Sun NF, Liu ZA, Huang WB, Tian AL, Hu SY (2014). The research of nanoparticles as gene vector for tumor gene therapy. Crit Rev Oncol Hematol..

[13] Vázquez E, Cubarsi R, Unzueta U, Roldán M, Domingo-Espín J, Ferrer-Miralles N (2010). Internalization and kinetics of nuclear migration of protein-only, arginine-rich nanoparticles. Biomaterials..

[14] Unzueta U, Céspedes MV, Ferrer-Miralles N, Casanova I, Cedano J, Corchero JL (2012). Intracellular CXCR4^+^ cell targeting with T22-empowered protein-only nanoparticles. Int J Nanomedicine..

[15] Trang P, Wiggins JF, Daige CL, Cho C, Omotola M, Brown D (2011). Systemic delivery of tumor suppressor microRNA mimics using a neutral lipid emulsion inhibits lung tumors in mice. Mol Ther..

[16] Hsu SH, Yu B, Wang X, Lu Y, Schmidt CR, Lee RJ (2013). Cationic lipid nanoparticles for therapeutic delivery of siRNA and miRNA to murine liver tumor. Nanomedicine..

[17] Balhorn R (2007). The protamine family of sperm nuclear proteins. Genome Biol..

[18] He HN, Ye JX, Liu EG, Liang QL, Liu Q, Yang VC (2014). Low molecular weight protamine (LMWP): a nontoxic protamine substitute and an effective cell-penetrating peptide. J Control Release..

[19] Martins RP, Ostermeier GC, Krawetz SA (2004). Nuclear matrix interactions at the human protamine domain: a working model of potentiation. J Biol Chem..

[20] Cornetta K, Anderson WF (1989). Protamine sulfate as an effective alternative to polybrene in retroviral-mediated gene-transfer: implications for human gene therapy. J Virol Methods..

[21] Amand HL, Rydberg HA, Fornander LH, Lincoln P, Nordén B, Esbjörner EK (1818). Cell surface binding and uptake of arginine and lysine-rich penetratin peptides in absence and presence of proteoglycans. Biochimica et Biophysica Acta (BBA)-. Biomembranes..

[22] Liu C, Yu W, Chen Z, Zhang J, Zhang N (2011). Enhanced gene transfection efficiency in CD13-positive vascular endothelial cells with targeted poly (lactic acid)–poly (ethylene glycol) nanoparticles through caveolae-mediated endocytosis. J Control Release..

[23] Tijsterman M, Plasterk RH (2004). Dicers at RISC: the mechanism of RNAi. Cell..

[24] Hannon GJ (2002). RNA interference. Nature..

